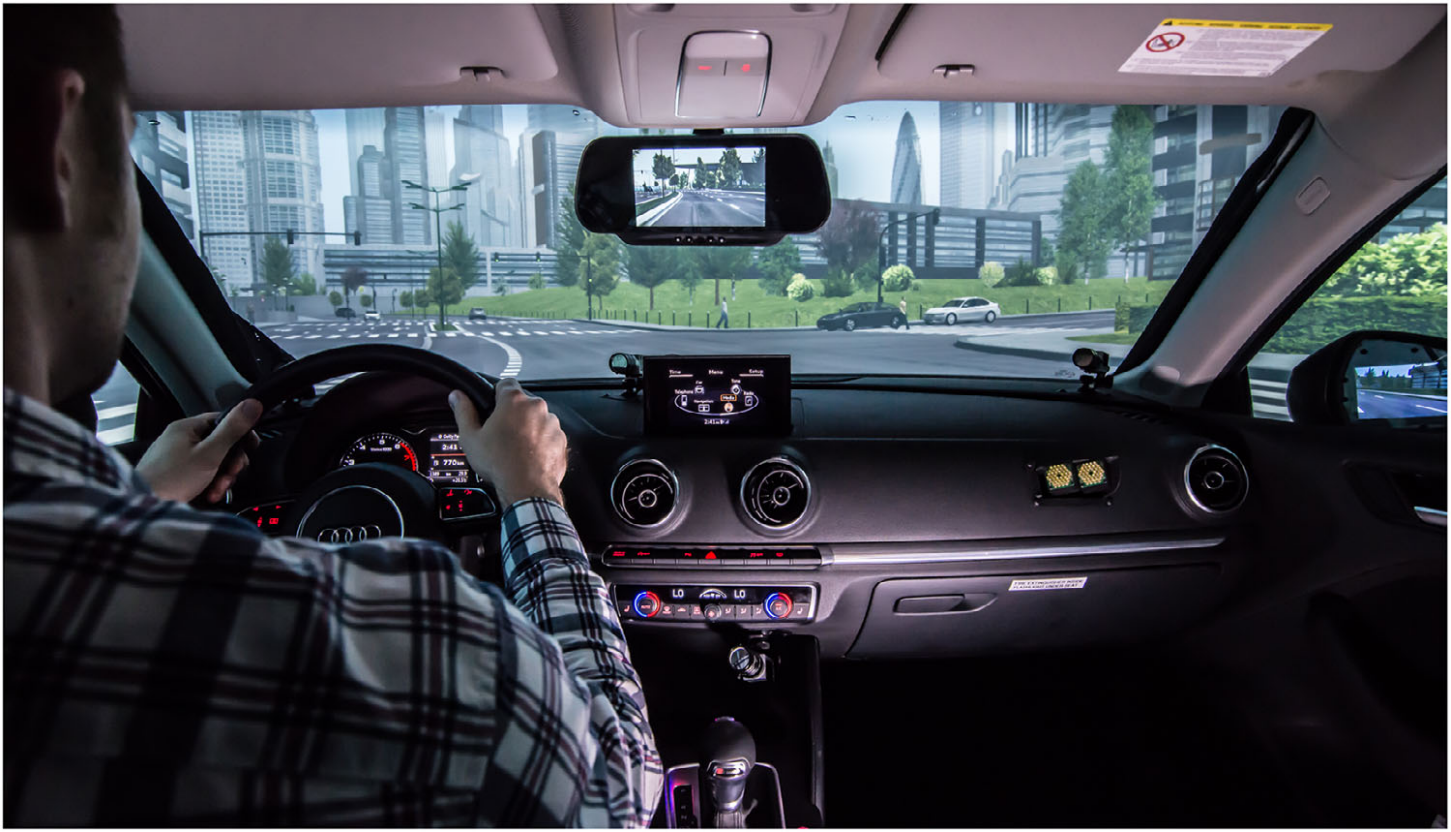# Driving Performance as a Marker of Cognitive Status: A Machine Learning‐ based Approach

**DOI:** 10.1002/alz70858_106244

**Published:** 2025-12-26

**Authors:** Gelareh Hajian, Bing Ye, Elaine Stasiulis, Mark J. Rapoport, Gary Naglie, Jennifer L. Campos, Alex Mihailidis

**Affiliations:** ^1^ KITE ‐ Toronto Rehabilitation Institute, University Health Network, Toronto, ON, Canada; ^2^ University of Toronto, Toronto, ON, Canada; ^3^ Baycrest Health Sciences, Toronto, ON, Canada; ^4^ Sunnybrook Health Sciences Centre, Toronto, ON, Canada; ^5^ Toronto Rehabilitation Institute, Toronto, ON, Canada

## Abstract

**Background:**

Driving is essential for maintaining independence and social engagement in many older adults, serving as both a practical mode of transportation and a symbol of autonomy. Aging, however, is associated with cognitive changes and increased risk of cognitive impairment (e.g. mild cognitive impairment and dementia). These changes in cognition can affect the complex skills required to drive and previous studies have suggested that changes to driving behavior can serve as an early marker of cognitive decline. This study aimed to determine whether machine learning can detect cognitive status (cognitively impaired vs. cognitively unimpaired) based on driving performance.

**Method:**

Eight cognitively healthy older adults and seven older adults with diagnosed cognitive impairments (mild cognitive impairment and very mild dementia) drove through various everyday scenarios in a high‐fidelity driving simulator. Driving performance metrics such as steering wheel angles, lane deviation, acceleration, and braking intensity were analyzed. Statistical features, including means, standard deviations, ranges, and cumulative changes, were extracted using sliding windows. A Random Forest model was developed as the primary predictive tool to detect cognitive status, given its ability to handle non‐linear relationships, and feature interactions. Model performance was compared with other machine learning models. Feature importance in the Random Forest model was assessed by evaluating each feature's contribution to reducing impurity during tree construction. A refined Random Forest model trained with the top five features was also evaluated.

**Result:**

The Random Forest model achieved the highest accuracy (66.67%) using all features, followed closely by Gradient Boosting (66.07%), with K‐Nearest Neighbors (63.10%), Decision Trees (62.50%), Support Vector Machine (60.12%), and Logistic Regression (58.93%). Feature importance analysis identified key predictors: cumulative and minimum acceleration, maximum and minimum steering wheel angle, and lane gap range. Using these top five features, the Random Forest model's accuracy improved to 70.83%.

**Conclusion:**

This study demonstrates the feasibility of using machine learning to classify cognitive status based on driving performance. Findings suggest that driving metrics have potential as a tool for detecting cognitive decline. In the future, we will extend this approach to detect pre‐clinical cognitive decline in individuals with subjective cognitive decline, assess AI's reliability, and support timely interventions.